# Too Many Is Too Bad: Long-Term Net Negative Effects of High Density Ungulate Populations on a Dominant Mediterranean Shrub

**DOI:** 10.1371/journal.pone.0158139

**Published:** 2016-07-07

**Authors:** Xavier Lecomte, José M. Fedriani, Maria C. Caldeira, Adelaide S. Clemente, Alessandro Olmi, Miguel N. Bugalho

**Affiliations:** 1 Forest Research Center (CEF), School of Agriculture, University of Lisbon, Lisbon, Portugal; 2 Centre for Applied Ecology (CEABN-InBio), School of Agriculture, University of Lisbon, Lisbon, Portugal; 3 Estacion Biológica Doñana, CSIC, Seville, Spain; 4 Centre for Ecology, Evolution and Environmental Changes (cE3c), Faculty of Sciences, University of Lisbon, Lisbon, Portugal; 5 Faculty of Agronomical Sciences, University of Florence, Florence, Italy; Instituto de Biología Molecular y Celular de Plantas, SPAIN

## Abstract

Plant–animal interactions imply costs and benefits with net balance depending on interacting species and ecological context. Ungulates, in particular, confer costs (e.g., plant leaf consumption, flower bud predation) and benefits (e.g., plant overcompensation, seed dispersal) to plants. Magnitude of costs and benefits may be altered by habitat management or ecological conditions favoring high density ungulate populations. Little is known however on whether plant costs or benefits predominate over the years, or the long-term outcomes of plant-animal interactions in habitat types sustaining high density ungulate populations. We investigated how high density ungulate populations alter plant costs and benefits by quantifying ungulate long-term effects on the shrub *Cistus ladanifer* (Cistaceae) individual size, seed weight and number, seed bank, and population density, through a 12-year ungulate exclusion experiment in a Mediterranean scrubland. We monitored plant size and flower buds in plants exposed or protected from ungulates and number of developed capsules and seeds consumed (potential seed dispersal) by ungulates during three reproductive seasons. We found that ungulates negatively affected shrub size and led to a dramatically decline of shrub reproductive structures and seed production, affecting the plant reproductive cycle. Number of buds was 27 times higher and number of developed seed 5 times higher in ungulate-excluded as compared to ungulate-exposed plots. After 9 years of ungulate exclusion, the *C*. *ladanifer* seed bank was 2.6 times higher in ungulate-excluded plots. The population density of *C*. *ladanifer* was 4 times higher in ungulate-excluded plots. Our long-term experiment showed that high density ungulate populations can alter plant-animal interactions by reducing plant benefits and increasing plant costs.

## Introduction

Species interactions are often described as either antagonistic or mutualistic, even though most of them correspond to a mixture of conflicting and overlapping interests, potentially being positive or negative for the participants depending on the ecological context [1–4]. Human induced changes can alter the biotic and abiotic context and be a major driver of shifts in sign and magnitude of species interactions [5]. Hunting, for example, is known to alter plant-mammal and plant-insect interactions [6, 7]. Also, habitat management practices (e.g. limiting culling policies, food supplementation) may favor the increase of animal populations such as ungulate herbivores which can affect the ecology of ecosystems and species interactions [8, 9]. Ungulate herbivores, in particular, confer costs (e.g. plant leaf consumption, flower bud predation) [10, 11] and benefits (e.g. plant overcompensation, seed dispersal) [12, 13, 14] to plants. The magnitude of such costs and benefits however may be altered by the ecological conditions namely ungulate population densities. For example, exclusion of large herbivores resulted in a shift from mutualistic to antagonist interactions in African tree-defender ants and acacias [15]. Conversely, high density ungulate populations resulting from favorable land use changes (e.g. increase of suitable woodland habitat following land abandonment) together with lack of predators and limited culling policies may affect the whole ecology of ecosystems and of species interactions [8, 9, 16, 17]. We are far, however, from understanding how high density ungulate populations may alter the patterns, mechanisms and outcomes of plant-animal interactions [5, 18]. One of the main obstacles to such an understanding is the lack of well-designed long-term field experiments allowing rigorous estimates of the effects of vertebrate herbivores on plants, both at the individual and the population levels. Because species interactions are critical for ecosystem functioning and ecosystem services delivery [5, 9, 19] further research on how human induced changes leading to high population densities of ungulates may alter plant-herbivore interactions, is clearly needed.

Ungulate herbivores consume plant leaves and often flower buds (herbivory costs) as well as fully-developed fruits comprising viable seeds (seed dispersal benefits) [12, 20]. The nature of such two-phase plant-ungulate interactions is expected to be mostly antagonistic if, in the long-term, herbivore populations affect negatively fruit production or exert too strong bud predation leading to too few, if any, seeds completing their development and being dispersed. For instance, deer herbivory (*Odocoileus hemionus* and *Cervus elaphus*) in a North American ponderosa pine forest reduced biomass and reproductive success of the shrub *Ceanothus fendleri* by 92% and 85%, respectively [11]. Likewise, herbivory by a high population density of red deer in the Greater Yellowstone ecosystem, USA, practically eliminated seed production of several Rosaceae and Elaeagnaceae shrubs [10]. Conversely, different studies have shown that ungulates can act as effective seed dispersers [12, 21, 22].

Furthermore, changes in fruit production due to leaf herbivory or direct bud or mature seed predation and potential seed dispersal, are likely to alter soil seed banks [23, 24], which are crucial determinants of the dynamics of many plant populations [25]. Long-term studies accounting for critical plant performance components (e.g. seed set, seed bank, seedling numbers) and changes in plant population density are thus needed for comprehensively understanding the long-term ecological effects of high density ungulate populations on the nature of plant-animal interactions.

In this study, we experimentally investigate the long-term effects of high-density populations of ungulates (red deer *Cervus elaphus* and fallow deer *Dama dama*) on plant size and several sequential reproductive components of *Cistus ladanifer* L. (Cistaceae), a seed-bank forming Mediterranean shrub, through an ungulate exclusion experiment. To identify the extent to which ungulate acted mostly as predators (antagonistic) or as potential seed dispersers (mutualistic), we estimated, separately, how long-term herbivory affected 1) plant size and subsequent flower-bud and seed production and 2) numbers of developing flower-buds and fully developed fruit capsules and 3) seed bank in ungulate-excluded (fenced) and ungulate-exposed (unfenced) plots. We hypothesized strong deer effects on *C*. *ladanifer* size, associated with effects on seed production, and direct predation of flower-buds and developed fruits. Furthermore, we expected that in the long-term ungulate herbivory would limit *C*. *ladanifer* population density. To evaluate this prediction, we also compared 4) adult shrub densities in ungulate-exposed and ungulate-excluded plots after 12-years of experimental exclusion.

## Materials and Methods

“Study area is privately owned. We had authorization of the landowner, Fundação da Casa de Bragança, for conducting research in the study area. Research activities did not involve endangered or protected species and did not require any specific authorization to be conducted. "

### Study site

The study area is located in Tapada Real de Vila Viçosa (Tapada de Baixo) in southeast Portugal (38°48’N, 07°24’W). This is a 900 ha enclosed estate, dominantly covered by cork (*Quercus suber* L.) and holm (*Q*. *ilex* ssp. *rotundifolia* Lam.) oak, exploited for cork and primarily managed for deer hunting (e.g. food-supplementation, selective culling). The climate is typically Mediterranean, characterized by hot and dry summers and cool and wet winters. Mean annual precipitation is 585.3 mm mainly falling between October and May. The mean annual temperature is 15.9°C with a maximum of 31.1°C (in July) and a minimum of 5.8°C (in January) (Évora meteorological station, 1981–2010, http://www.ipma.pt, accessed in January 2013).

The evergreen cork and holm oak woodland is relatively open (30 to 50 trees per ha), with an almost mono-specific understory of the shrub *C*. *ladanifer* L. interspersed with annual grasslands [26]. The site is browsed by herbivore ungulates, red deer (*Cervus elaphus*) and fallow deer (*Dama dama*) which, due to a limited culling policy and supplementary feeding in years of lower food availability, have been maintained in the study area at population densities of 0.35 and 0.1 deer per ha respectively. Although not uncommon in Iberian Peninsula hunting estates, such population densities are generally considered as high [27, 28]. Few wild boars (*Sus scrofa*, L.) occurred at the beginning of the experiment, although they have locally thrived in recent years.

*Cistus ladanifer*, a woody perennial shrub, common to the western Mediterranean Basin, including southern Europe and northern Africa, is an obligate seeder [29]. Fruits are globular lignified capsules which may contain between 500 to 1000 hard seeds. Flowering occurs between March and April followed by fruit maturation between May and July. Release of seeds starts in mid-summer and extends until the end of summer to the beginning of autumn [30, 31]. Evergreen or summer semi-deciduous shrubs, such as *C*. *ladanifer*, can be an important source of protein for herbivores during winter (January to April) and during the late Mediterranean summer to beginning of autumn (July to October), when most grasses are senescent and of low nutritive value [32, 33]. Thus, throughout the year, deer eats *C*. *ladanifer* young shoots and leaves but also buds, flowers and developed capsules [32, 34] promoting dispersion and germination of *C*. *ladanifer* seeds [12].

### Experimental design

In July 2001, 5 blocks of paired fenced (ungulate-excluded) and unfenced (ungulate-exposed) plots of 25 m × 25 m were randomly established in homogeneous areas of grassland, with no presence of *C*. *ladanifer* adults or juveniles. *C*. *ladanifer* shrubs had been previously cleared from these areas as part of common management practices in the estate. In Mediterranean cork and holm oak woodlands, shrubs are mechanically cleared each 4 to 7 years, rotationally in different locations, to prevent wildfires. Fences were 2.20 m tall for ensuring ungulate exclusion. Distance between paired (fence and unfenced) plots was approximately 25 m and between adjacent pairs between 250 m to 400 m. One open plot was lost in July 2004 and, thus, we considered data from the remaining four pairs of plots

### Long-term effects of ungulates on size of *C*. *ladanifer*

For estimating overall ungulate herbivory pressure (i.e. on both vegetative and reproductive plant structures), we compared the volume of *C*. *ladanifer* shrubs in ungulate-excluded and ungulate-exposed plots. To this end, we randomly selected 15 (in 2007) and 4 to 6 (in 2008 and 2013, respectively) *C*. *ladanifer* individuals in ungulated-exposed (open) and ungulate-excluded plots (fenced). Height and diameter of canopy projection of each sampled individual shrub was measured and shrub volume was estimated assuming the shape of an elliptical cone given by:

$$V=\left(\frac{1}{3}\right)\times \pi \times \frac{D1}{2}\times \frac{D2}{2}\times H$$

in which V is the volume of the shrub, D1 is maximum diameter of shrub canopy projection, D2 is diameter perpendicular to D1 and H is maximum shrub height.

To rule out the possibility that potential differences in plant individual size (shrub volume) and flower bud production between ungulate exclusion treatments were related to shrub age differences, we randomly selected and cut to ground level 4 shrubs in each plot in April 2008. Age of each individual shrub was then estimated through annual growth rings count. Growth rings were counted at the base of the trunk after buffing the surface with a high grade sand paper [35]. We found no significant differences in age between *C*. *ladanifer* individuals in ungulate excluded (6.06 ± 0.11 years; mean ± s.e.m.) vs. ungulate exposed plots (5.71 ± 0.14 years; Mann Whitney U-test, U = 93, P = 0.127).

#### Effects of ungulates on buds and developed capsules

We monitored 60 and 60 (2007), 19 and 16 (2008), 40 and 12 (2013) *C*. *ladanifer* individuals in ungulate-exposed and ungulate-excluded plots, respectively. We recorded and compared in April of each year the number of buds (plus open flowers) remaining in ungulate-exposed and ungulate-excluded plots. To estimate the potential of ungulates to act as seed dispersers, during July and October of 2007 (i.e. when most capsules were ripe), we estimated the percentage of developed capsules removed by ungulates relative to the number of available buds or capsules in April and July, respectively. Although we did not directly confirm that ingestion of fully developed seeds leads to their dispersal, there is strong evidence that ungulates, and deer in particular, can act as seed dispersers when ingesting mature plant fruits (e.g. [12]). Because of the lack of teeth in front upper jaw, browsing marks left by deer (i.e. ragged edge on damaged stems) can be easily distinguished from browsing damage left by other herbivores such as rabbits, hares or voles (i.e. leave sharp-angled, knife-like cut on ends of stems) which sporadically occur in the area. Aborted fruits remained on *C*. *ladanifer* branches and were easily distinguished from normally developed fruits in ungulate-excluded and ungulate-exposed plots. Thus we only recorded differences between treatments in normally developed flower-buds and developed capsules at each sampling date.

#### Effects of ungulates on seed numbers, seed weight and germination

We randomly selected 5 shrubs per plot and collected 6 and 3 fruit capsules from each of these shrubs in July 2007 and July 2008, respectively. We collected *C*. *ladanifer* capsules without any signs of predation in July when seeds are mature. Capsules were then conserved at 4°C in a freezer. We estimated seed weight and number of seeds per capsule by weighing 100 seeds that were previously oven-dried at 60°C during 72 h. These seeds were randomly taken from 20 capsules collected in ungulate-exposed and 20 capsules collected in ungulate-excluded plots. Overall, 120 and 60 capsules per treatment were collected in 2007 and 2008, respectively.

For estimating seed germination we made composite seed samples per shrub using seeds from capsules collected in July 2008. Four replicates of 25 seeds each were then taken from each composite sample (2000 seeds per treatment overall) and seeds were placed in an oven at 100°C during 5 minutes to break dormancy [36]. Seeds were then distributed on filter paper disks (Whatman #1, n° 1001 125, GE Healthcare, Buckinghamshire, UK), randomly placed on modified Jacobsen individual apparatus trays [37] in a germination incubator. The germination incubator was kept at a constant temperature (20°C) and under a 16 h-light photoperiod. Photosynthetic Photon Flux Density of 140 micromolm^-2^.s^-1^ was provided and measured with a Quantum Radiometer (Model LI-170, Li-Cor, Lincoln, N.E., USA). Every 3 days we counted and removed germinated seeds and seeds damaged by fungi from trays to avoid contamination of other seeds. Seeds were recorded as germinated as soon as the radicle emerged. The experiment was conducted over 105 days after which period no further germination was observed.

#### Effect of ungulates on soil seed bank

Soil seed bank was estimated by the seedling emergence method [38]. Soil samples were collected at the end of October 2010 (after 9 years of ungulate exclusion) at the beginning of the germination period [30]. We randomly collected 18 soil cores per plot (0.05 m in diameter × 0.05 m in height) in each of the ungulate-excluded and ungulate-exposed plots. Samples were kept in the dark at 10°C, for 5 days, until the beginning of the emergence assay. We randomly placed homogenized soil samples in polyethylene containers (17 cm x 12 cm x 3 cm) and over a 1.5 cm bed of sterilized sand to allow seed germination. Soil was maintained moist, near field capacity, by an automatic irrigation system. We then identified, counted and removed emerged *C*. *ladanifer* seedlings from the containers, immediately after germination. Germinated seedlings of herbaceous species were removed to avoid competition with *C*. *ladanifer* seedlings. Germination trials lasted for 75 days until no more seed germination was observed.

#### Long-term ungulate induced changes in the population density of *C*. *ladanifer*

To estimate the long-term effect of ungulate activity on the population density of plants of *C*. *ladanifer*, we compared changes in shrub density in ungulate-exposed and ungulate-excluded plots after 12-years of experimental exclusion of ungulates. To this end, we randomly selected 18 (2 x 4 m) sub-plots within each plot (25 x 25 m) and recorded all individual adults of *C*. *ladanifer* during spring of 2007 and 2013. No *C*. *ladanifer* individuals occurred in any plot at the beginning of the experiment in 2001 (thus initial plot conditions could not affect our results). In 2007 all *C*. *ladanifer* individuals were alive both in ungulate-exposed and ungulate-excluded plots. In 2013, however, dead individuals occurred in the plots and their numbers were also recorded.

### Statistical analysis

Data on *C*. *ladanifer* bud and capsule numbers, shrub volume, seed weight and number, percentage of germination, number of emerged seedlings, and adult density were analyzed fitting generalized linear mixed models using Proc Glimmix in SAS [39]. The effects of ungulate exclusion and year, as well as their interaction, were specified in the models as fixed effects, whereas the experimental plot and replicate (nested within plot) were included as random factors. A significant interaction between ungulate exclusion and year would indicate temporal inconsistency in the effect of herbivores on *C*. *ladanifer* performance. For proportions of seed germination, we specified in the corresponding models binomial error and logit-link function (see [39]). Because of high number of zero values in count response variables such as the number of emerged seedlings from soil samples and the number of adult *C*. *ladanifer*, we specified negative binomial (instead of Poisson) error and log-link function. For shrub volume we specified normal error and identity-link function. When the interaction between ungulate exclusion and year was significant, we performed tests for the effect of a given factor at the different levels of the other factor (“tests of simple main effects”), using the SLICE option in the LSMEANS statement of the MIXED procedure [39]. For comparing number of dead individual *C*. *ladanifer* shrubs between treatments in 2013 we used Mann-Whitney tests [40].

## Results

### Effects of ungulates on *C*. *ladanifer* size and number of flower buds

**Fig 1:**
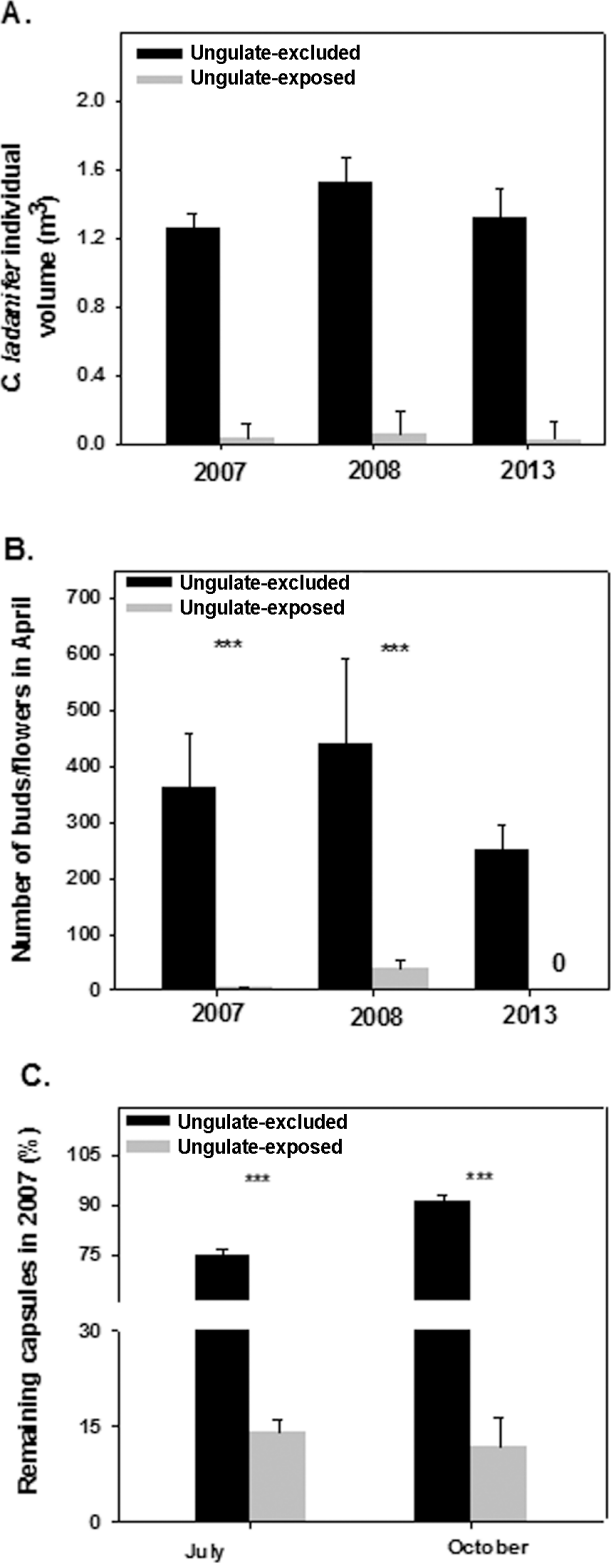
**Model-adjusted means (± 1 SE) of A) *Cistus ladanifer* volume per individual in 2007, 2008 and 2013 B) Numbers of flower buds (plus open flowers) produced during 2007, 2008, and 2013 seasons in ungulate-excluded and ungulate-exposed (open) plots and C) Percentages of remaining capsules in July (regarding flower bud number in April) and October 2007 (regarding capsule number in July).** (***, *P* < 0.001)

*Cistus ladanifer* volume in ungulate-excluded plots was on average 33.6 times larger as compared with shrubs within ungulate-exposed plots (F_1,198_ = 197.09, *P* < 0.0001; Fig 1A). The differences were consistent among the three years, as indicated by the non-significant interaction between ungulate exclusion and year (F_2,198_ = 0.61, *P* = 0.542). We did not find significant differences in *C*. *ladanifer* volume among years (F_2,198_ = 0.98, *P* = 0.378; Fig 1A).

Our generalized linear mixed model indicated that, once the effect of the random factor (i.e. plot) was controlled for, year had a significant effect as main factor on the number of *C*. *ladanifer* flower-buds (F_1,148_ = 32.85, *P* < 0.0001) being, on average, over 3-fold higher in 2008 than in 2007 (Fig 1B). As expected, ungulate exclusion also had a strong significant effect on number of flower buds (F_1,148_ = 269.51, *P* < 0.0001) being, on average, 27.3-fold higher in ungulate-excluded as compared to ungulate-exposed plots (Fig 1B). There was also a significant interaction between ungulate exclusion and year (F_1,148_ = 22.31, *P* < 0.0001), indicating that the effect of ungulate exclusion on *C*. *ladanifer* flower bud number was stronger during 2007 than during 2008 (Fig 1B). Given that during 2013 no individuals in ungulate-exposed plots (n = 40) produced buds, 2013 data was analyzed separately. Whereas all 12 individuals within ungulate-excluded plots produced abundant buds (Fig 1B), none individual in the ungulate-exposed plots produce any bud (χ^2^ = 52.0, df = 1, *P* < 0.0001). Thus, 2013 data confirmed the trend revealed in the previous two years, i.e. strong negative ungulate effects on number of *C*. *ladanifer* flower-buds.

### Effects of ungulates on developed capsules

Our monitoring of bud fate during 2007 revealed that, as expected, the percentage of buds counted in April and remaining in July as developed capsules was 5.3-fold higher in ungulate-excluded plots as compared to ungulate-exposed plots (F_1,82_ = 347.75, *P* < 0.0001; Fig 1C). Similarly, the percentage of capsules counted in July that remained in October was 7.7-fold higher in ungulate-excluded plots as compared to ungulate-exposed plots (F_1,68_ = 130.87, *P* < 0.0001; Fig 1C). Overall, these results show sizable levels of ungulate consumption of developed capsules, indicating that they may act as potential seed dispersers.

### Effects of ungulates on seed weight and number, soil seed bank and germination

**Fig 2:**
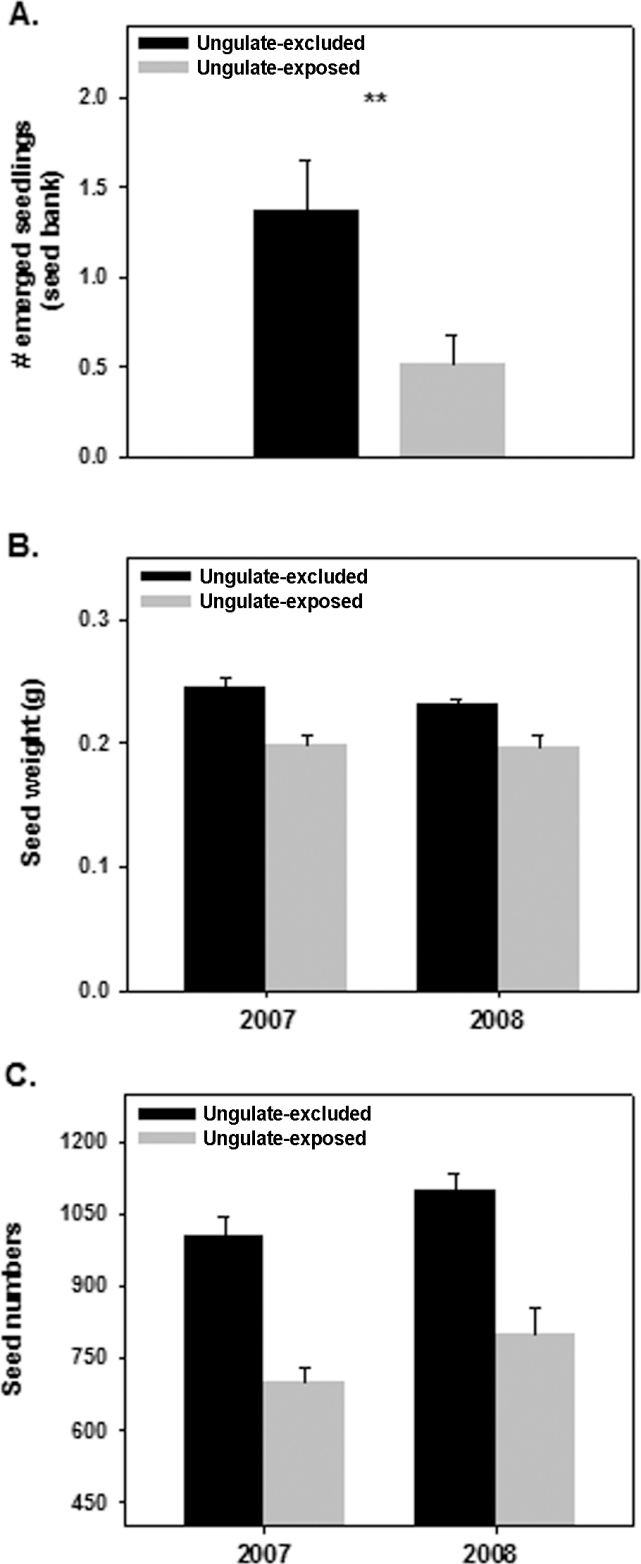
**Model-adjusted means (± 1 SE) of A) Number of *C*. *ladanifer* seedlings emerged from soil seed bank samples B) Seed weight and C) Seed numbers from capsules collected in ungulate-excluded and ungulate-exposed (open) plots** (**, *P* < 0.01; ***, *P* < 0.001; and ns, not significant [*P* > 0.05]).

Our mixed model indicated that, once the effect of plot was corrected for, seedling emergence in soil samples from ungulate-excluded plots was 2.6 times higher than in ungulate-exposed plots (F_1,75_ = 8.15, *P* < 0.01; Fig 2A). Our chamber experiment revealed that, seed germination did not vary between ungulate-excluded and ungulate-exposed plots (F_1,143_ = 0.45, *P* = 0.502; Fig 2B) in spite of seed weight being significantly higher in both years in ungulate excluded plots (seed weight in ungulate-excluded and ungulate-exposed plots, respectively: 0.245 ± 0.007 vs. 0.198 ± 0.008 mg in 2007 and 0.231 ±0.004 vs. 0.197 ± 0.009 mg in 2008, F_1,119_ = 11.19, *P* < 0.001) (Fig 2C). Overall, these results showed a higher seedling emergence in ungulate-excluded soil samples, in spite of no differences in seed germination between treatments. These results are also consistent with a higher number of seeds per capsule found in ungulate-excluded plots (seed numbers per capsule in ungulate-excluded and ungulate-exposed plots, respectively: 1006.20 ± 36.63 vs. 701.88 ± 29.85 seeds per plot in 2007 and 1100.07 ± 33.32 vs. 798.45 ± 57.61 seeds per plot in 2008, F_1,307_ = 30.41, *P* < 0.001) (Fig 2C).

### Effects of ungulates on the population density of *C*. *ladanifer*

**Fig 3:**
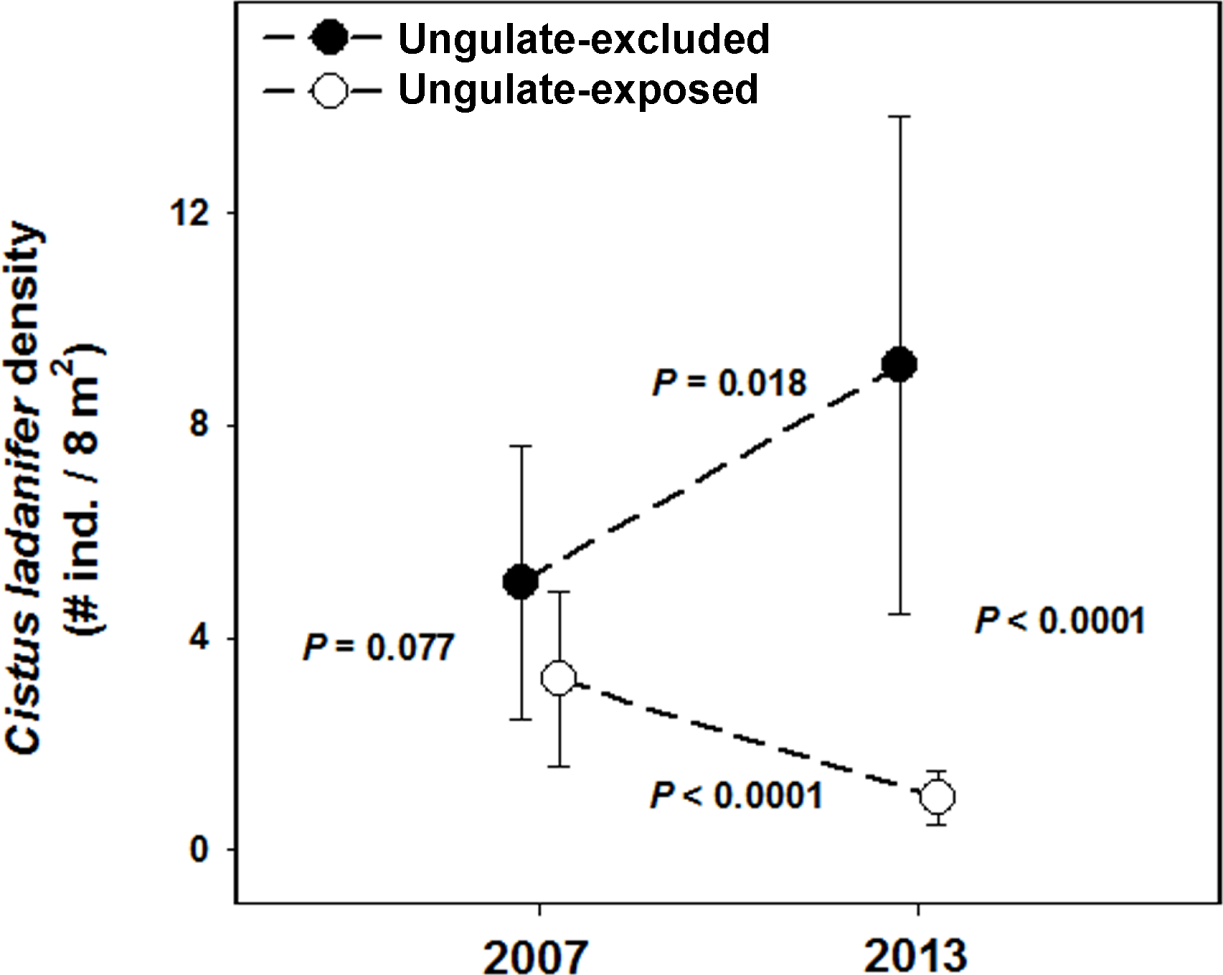
Changes in *C*. *ladanifer* population density (shrubs per 8 m^2^) in 2007 and 2013 in the ungulate-excluded and ungulate-exposed plots. Because the interaction between ungulate exclusion and year was significant, we report the *P*-values of the tests for the four simple main effects involved in the interaction.

Although no *C*. *ladanifer* individual occurred in our experimental plots in 2001, colonization started soon after the field experiment was initiated (Authors personal observation). Ungulate exclusion had a strong significant effect as main factor on *C*. *ladanifer* density (F_1,281_ = 51.86, *P* < 0.0001)which was, on average, over 3.8-fold higher in ungulate-excluded as compared with ungulate-exposed plots (Fig 3). Year (2007 and 2013) did not have an effect as main factor (*P* = 0.109), but it showed a significant interaction with ungulate exclusion (F_1,281_ = 22.89, *P* < 0.0001) indicating that the sign and/or strength of its effect on the population density of *C*. *ladanifer* varied between ungulate-exposed and ungulate-excluded plots (Fig 3). Specifically, tests of slices revealed that whereas *C*. *ladanifer* population density in ungulate-excluded plots increased during the five experimental years (F_1,281_ = 5.69, *P* < 0.05), it clearly shrank in ungulate-exposed plots during such time period (F_1,148_ = 18.37, *P* < 0.0001; Fig 3). Furthermore, whereas no *C*. *ladanifer* individuals occurred in any of the plots in 2001, and whereas *C*. *ladanifer* population density did not significantly differ between ungulate-exposed and ungulate-excluded in 2007 (F_1,281_ = 3.14, *P* = 0.077), at the end of the experiment *C*. *ladanifer* population density was significantly lower in ungulate-exposed plots (F_1,281_ = 67.40, *P* = 0.0001; Fig 3). Additionally a higher number of dead individuals was recorded in ungulate exposed plots in 2013 (4.57 ± 0.55 vs. 7.38 ± 0.79 dead individuals/plot, in ungulate-exposed vs. ungulate-excluded plots, respectively, *P* < 0.0001, Mann-Whitney U test, U = 58.5).

## Discussion

Plant-animal interactions imply both costs and benefits for the participants [2, 3] that may ultimately affect their population densities [41, 42]. Our study showed that high density populations of ungulate herbivores can have a drastic negative effect on plant size and number of *C*. *ladanifer* flower-buds produced thus, limiting the availability of ripe fruits holding fully developed seeds that could be disseminated into the soil seed bank. The effects of such dramatic decline of shrub reproductive structures, translated along the plant reproductive cycle and led to reduced fruit set, limited soil seed bank, ultimatelyaffecting the population density of *C*. *ladanifer*. The long-term net balance between ungulate herbivory and its effects on number of flower buds and potential seed dispersal was thus highly negative for the plant, as illustrated by the strong decrease of *C*. *ladanifer* density after 12 years. These results highlight the potential of high density ungulate populations, favored by habitat management practices such as limited culling policies and food supplementing, to alter the nature of species interactions [5, 19, 43].

### Individual and long-term population effects of *C*. *ladanifer*—ungulate interactions

Although strong negative effects of ungulates on plant reproductive success [44–47] or on growth and survival [48–51] have been often documented, few studies have assessed the population consequences of these interactions (but see [51]). Our long-term ungulate-exclusion revealed that cumulative effects of ungulates ultimately led to a decline of *C*. *ladanifer* population density, possibly by decreasing the seed bank. Although depletion of seed banks, as affected by heavy grazing, has been shown in grasslands [52, 53] less is known for woody plant communities, for which browsing and grazing have been mainly shown to alter plant succession [53, 54]. In our study, we have shown that ungulate herbivory on *C*. *ladanifer* led to a noticeable soil seed bank depression after 9 years. Given that persistent soil seed banks are especially critical in highly climatic variable ecosystems [25], such as Mediterranean ecosystems [55], this is likely to negatively affect *C*. *ladanifer* dynamics and resilience, which seeds can persist viable in the soil seed bank for 6 to 7 years [56, 57]. Furthermore, although herbivores can confer plants with benefits such as over-growth [47, 58] or seed dissemination [12, 51], very few studies have assessed separately negative and positive effects of herbivores on plants. Here, we have shown strong negative effects of ungulate herbivores on *C*. *ladanifer* plant size and reproductive structures, but also considerable ungulate ingestion of ripe capsules comprising fully developed seeds, which were likely to being dispersed [12]. Our results, suggest that ungulate herbivory seems to have overridden potential positive effects of ungulates on *C*. *ladanifer* seed dissemination. Moreover, our conclusions are probably conservative as our experimental setting prevented any arrival of dispersed seeds in the ungulate-excluded plots but not in the ungulate-exposed plots.

The strong net negative cumulative effect of ungulates on the population density of *C*. *ladanifer*, may have been exacerbated by synergistic effects of ungulate herbivory and drought [59]. Indeed, 2012 was an extremely dry year [60] and the higher mortality of *C*. *ladanifer* observed in ungulate-exposed plots in subsequent year may have resulted from herbivory and drought interactions. Interestingly, our results also suggest a considerable lag effect of herbivory on *C*. *ladanifer* population density. These results highlight the importance of long-term field experiments when investigating the population outcomes of plant-animal interactions (e.g. [44]). Results also emphasize that only long term studies, such as ours, can properly capture the effects of high inter-annual climatic variability, such as that typical of Mediterranean environments, when investigating species interactions.

### Context-dependency of plant-ungulate interactions and community-level effects

Variation in the strength and sign of plant-herbivore interactions are likely to occur as a result of changes in the ecological context (e.g. [1–3]). The intensive ungulate consumption of *C*. *ladanifer*, a species often considered as unpalatable [61], during this and previous studies [32, 62] is likely to be related to the generalist feeding habits of target consumers, which tend to feed on woody and grass species according to plant food availability [63]. The high ungulate population densities during the period of our study probably enhanced food competition and forced animals to become less selective, leading to a higher consumption of *C*. *ladanifer* [61]. Also, when availability and quality of grasses is low, browsing pressure on *C*. *ladanifer* is likely to increase and to occur early in the season, as compared with years of higher availability of grasses [32, 64]. This will exacerbate negative effects on shrub plant size and number of flower buds produced. Further long-term research to unravel the extent to which variation in the strength and sign of *C*. *ladanifer*-ungulate interactions relate to bud production or to third-party factors such as availability of alternative plant food, such as grasses, is certainly needed.

The intensive interaction between high-density ungulate populations and *C*. *ladanifer* can, in addition, cascade through the whole ecosystem [15, 65] affecting biodiversity and ecosystem services delivery. For example, because ungulates can dramatically reduce *C*. *ladanifer* population densities, which is a shrub species that strongly competes with herbs and grasses in evergreen oak woodlands, in areas where ungulates are limiting the expansion of *C*. *ladanifer* populations, they may be indirectly favoring grass abundance and diversity [66]. Also, by decreasing shrub population densities, as well as individual shrub volume and thus biomass (Fig 1A), high density ungulate populations are likely to lessen the risk and severity of wildfires, an important ecosystem service in several human-shaped Mediterranean ecosystems [67].

Our long-term experimental study illustrates how high population densities of ungulates, favored either by habitat management (e.g. food-supplementation, under-harvesting) or land use changes that increase the availability of suitable habitat [68], may alter the balance between costs and benefits in plant-animal interactions. Further research on the community and whole ecosystem-level effects of over-abundant ungulate populations are certainly needed to forecast the outcomes of global change in species interactions and ecosystem ecology.
